# Constrained Active Fault Tolerant Control Based on Active Fault Diagnosis and Interpolation Optimization

**DOI:** 10.3390/e23080924

**Published:** 2021-07-21

**Authors:** Kezhen Han, Changzhi Chen, Mengdi Chen, Zipeng Wang

**Affiliations:** School of Electrical Engineering, University of Jinan, Jinan 250022, China; 202021100375@mail.ujn.edu.cn (C.C.); 202021200742@mail.ujn.edu.cn (M.C.); cse_wangzp@ujn.edu.cn (Z.W.)

**Keywords:** active diagnosis, active reconfiguration, constrained systems, fault tolerance, interpolation control, linear programming

## Abstract

A new active fault tolerant control scheme based on active fault diagnosis is proposed to address the component/actuator faults for systems with state and input constraints. Firstly, the active fault diagnosis is composed of diagnostic observers, constant auxiliary signals, and separation hyperplanes, all of which are designed offline. In online applications, only a single diagnostic observer is activated to achieve fault detection and isolation. Compared with the traditional multi-observer parallel diagnosis methods, such a design is beneficial to improve the diagnostic efficiency. Secondly, the active fault tolerant control is composed of outer fault tolerant control, inner fault tolerant control and a linear-programming-based interpolation control algorithm. The inner fault tolerant control is determined offline and satisfies the prescribed optimal control performance requirement. The outer fault tolerant control is used to enlarge the feasible region, and it needs to be determined online together with the interpolation optimization. In online applications, the updated state estimates trigger the adjustment of the interpolation algorithm, which in turn enables control reconfiguration by implicitly optimizing the dynamic convex combination of outer fault tolerant control and inner fault tolerant control. This control scheme contributes to further reducing the computational effort of traditional constrained predictive fault tolerant control methods. In addition, each pair of inner fault tolerant control and diagnostic observer is designed integratedly to suppress the robust interaction influences between estimation error and control error. The soft constraint method is further integrated to handle some cases that lead to constraint violations. The effectiveness of these designs is finally validated by a case study of a wastewater treatment plant model.

## 1. Introduction

Fault tolerance is already a common design property to be considered for most control systems. In terms of the system structure, faults can be classified as sensor faults, actuator faults, and component/parameter faults [1,2]. In general, the first two do not directly affect the intrinsic stability of the system, while the component faults tend to directly change the dynamic characteristics of the system. In the literature, the methods to handle these types of faults can be divided into active fault tolerant control (AFTC) and passive fault tolerant control (PFTC) [3]. PFTC draws on robust control theory to suppress the effects of faults, while AFTC uses fault information to adjust or reconfigure control actions to match the dynamics of the faulty system. Due to such matching adjustments, AFTC typically provides better reliability than PFTC. Many representative results can be found in the survey papers [4,5,6,7].

Recently, the design of optimal AFTC for systems with state/input constraints has been received a lot of attention. Unlike the design of unconstrained FTC, the design of constrained FTC has to take into account more requirements, including robust stability, feasibility, optimization efficiency, etc. Particularly, the faults occurring in constrained systems often cause constraint violations, and the unconstrained FTC designed without considering feasibility may result in an empty set of feasible solutions for a given control objective. This often further leads to the eventual loss of closed-loop stability. In the literature, some typical design methods for constrained FTC have been reported, such as Barrier Lyapunov function method [8], command governor [9,10], saturation control [11], model predictive control (MPC) [12], etc. Among these methods, the MPC-based FTC method is widely considered, since MPC has the inherent and flexible capacity to address constrained optimization problems. The representative studies include FTC based on min-max MPC [13], FTC based on explicit MPC [14], multi-actuator/sensor FTC based on set theoretic MPC [15,16,17,18], FTC based on dual model MPC [19,20], etc.

Most of the above mentioned MPC-based FTC designs are developed for actuator and sensor faults, whereas relatively few results are reported for component/parameter faults. Since the component faults often change the structural parameters of the system, determining the real-time operating mode of the system is a prerequisite for achieving fault tolerance. A common approach to this problem is to use multiple observers to first discriminate the fault modes, and then activate the corresponding control law of the isolated mode to achieve switching control reconfiguration [21]. Such an approach can be viewed as a passive fault diagnosis (PFD)-based AFTC scheme. Actually, due to the potential lack of diagnostically relevant information in the input–output data, the PFD method may fail to isolate a fault or may isolate a fault incorrectly. Moreover, for high-dimension systems, the multiple observers for parallel applications usually occupy a large amount of memory, and the involved modal discriminant optimization problem is generally computationally demanding. One promising way is to integrate the active fault diagnosis (AFD) methods into FTC, i.e., the AFD-based AFTC. The central idea in AFD is to design a small harmful test/auxiliary input signal that can ensure maximal or full separation among the model predictions corresponding to the different modes of operation [22]. According to different design methods of AFD, some representative results have been presented, such as AFTC based on Youla–Kucera parametrization [23], AFTC based on set detection and isolation [24], AFTC based on performance transformation [25], AFTC based on distributed fault isolation [26], etc.

The above studies have provided different ideas for the construction of AFD and AFTC. Inspired by these results, we find two more problems whose handling can be further improved:

(i) In terms of the implementation of AFD in AFTC, the test inputs used for modal separation are usually optimized online. For the small-scale systems, such computational requirements can be satisfied. However, as the number of system dimensions increases, the computational burden tends to become heavier, which often results in much longer delays of correct fault mode isolation. Recently, an effective solution was proposed in [27], where an implicit expression of the residual limit set is adopted and a constant auxiliary signal and the associated separation hyperplane used to separate the potential system modes are constructed offline. After a fault is detected, only the constant test signal is injected into the system and the current diagnostic observer. Then, the true system mode can be isolated by discriminating the position of the generated residuals in relation to the previously computed separation hyperplane. Given its advantages, such as simple implementation and fast isolation, this approach can provide an effective perspective for the design of control reconfigurations. Therefore, this paper will first attempt to adapt this active fault isolation approach to be integrated into the framework of AFTC to provide critical modal update information for timely regulation of constrained systems.
(ii) In terms of the design of constrained active reconfiguration FTC, most MPC-based methods need to solve computationally intensive optimization problems online. Generally, this often places stringent requirements on the system scale, sample interval, and hardware controller performance. As an alternative solution to constrained optimization, the interpolation control (IC) methods exhibit excellent features [28,29,30]. The main idea is to optimize an interpolation coefficient in real time based on the updated system states and use this coefficient to make a smooth convex combination of a outer controller and a inner controller. The outer controller is used to enlarge the controllable feasible domain, while the inner controller is used to satisfy the given control performance requirements. In general, the inner controller is optimally designed offline, while the outer controller is determined online simultaneously when the interpolation coefficient is optimized. This method of offline designing some parameters of the controller in advance helps to reduce the online calculation burden. Moreover, the optimized interpolation coefficient enables a smooth transition between the inner–outer controllers and ensures a fast convergence of the states to the set point under the constraints. In particular, the associated optimization problem belongs to standard linear programming (LP), which can be readily solved in the practical implementation. Given these characteristics, the IC-based optimization can provide a good compromise among computational load, feasible region size, performance, etc. Therefore, the development of the IC strategy to solve the constrained AFTC problem would be very promising. To the authors’ knowledge, no relevant results have been reported.

Motivated by the above observation, we seek to further push the development of the field of constrained FTC for component/actuator faults by proposing a new AFD-based interpolating FTC synthesis scheme. The central ideas of the technical route are: (1) the passive fault detection (FD) is firstly designed by using a diagnostic observer in the current mode; (2) after a fault is detected, the active fault isolation (FI) and mode identification are then achieved by using a constant test signal and a separated hyperplane; and (3) after the actual mode is isolated, the constrained AFTC is finally determined by virtue of optimizing the interpolation coefficient to combine the inner FTC and outer FTC. How to comprehensively solve the problems involved in this technical route is the main research content of this paper.

Compared with the recent results on constrained AFTC studies (e.g., [13,16,20]), our main contributions can be reflected in the follows aspects: (i) A new and efficient AFD-based AFTC approach for component/actuator faults is proposed. In this work, only one observer is applied in real time to achieve FD and FI, while most of the existing studies use multiple observers for online parallel diagnosis; the fault mode separation is achieved by using auxiliary signals and separating hyperplanes designed offline, rather than by solving receding horizon optimizations and set membership discriminations online; the real-time control reconfiguration-based AFTC is achieved by solving simple LP problems instead of solving quadratic or semi-definite positive programming problems. (ii) When designing diagnostic observers and FTCs, the interaction influences between estimation error and control error is further handled based on integrated design and constraint tightening so as to improve the robust feasibility of AFTC optimization algorithm. (iii) The soft constraints IC-based AFTC strategy is also designed to address some infeasible scenarios, such as, the deviation of states from the maximum controllable invariant set after fault isolation, or the constraints violation caused by some unanticipated factors.

The remainder of this paper is structured as follows. Section 2 provides the problem formulation. In Section 3, the proposed AFD-based interpolating AFTC scheme is explained in detail and an integrated algorithm is also given to summarize the involved offline design and online application steps. In Section 4, the algorithm verification is given. Some conclusion and future work are discussed in Section 5.

**Notation** **1.** *$diag\{X_{1},X_{2},X_{3}\}$ is a diagonal matrix with diagonal elements $X_{1}$, $X_{2}$, and $X_{3}$. $A^{T}P\left(*\right)=A^{T}PA$. $1_{m}$ is a m-dimensional column vector with all elements of 1, while $I_{m}$ is a m-dimensional unitary matrix. Let p∈P and q∈Q be two sets of $R^{n}$. Then, P⊕Q={p+q|p∈P,q∈Q} is the Minkowsi sum of two sets. For two sets satisfying Q⊂P, x∈P∼Q represents x∈P, but x∉Q. A polyhedron is the intersection of a finite number of open and/or closed half-spaces, and a polytope is a closed and bounded polyhedron.*

## 2. System Description and Problem Formulation

Consider the following uncertain discrete-time systems affected by unknown component faults, actuator faults and disturbances:

(1) $$\begin{array}{c} x_{k+1}=A^{l}x_{k}+B^{l}u_{k}+d_{k},\;y_{k}=Cx_{k}+v_{k} \end{array}$$

where $x_{k}\in X\subset R^{n}$ is the state vector; $u_{k}\in U\subset R^{n_{u}}$ is the actuator input vector; $d_{k}\in D\subset R^{n_{d}}$ is the unknown process disturbance vector; $v_{k}\in V\subset R^{n_{v}}$ is the unknown measurement disturbance vector; $y_{k}\in R^{n_{y}}$ is the measurement output vector. The matrices $A^{l}$, $B^{l}$ and *C* are constant and have appropriate dimensions. The index *l* is associated with the configuration in which the system is actually operating, i.e., $\left(A^{l},B^{l}\right)\in \left\{\left(A^{0},B^{0}\right),\;\left(A^{1},B^{1}\right),\;\cdots ,\left(A^{n_{f}},B^{n_{f}}\right)\right\},l\in \left[0,n_{f}\right]$. Without loss of generality, we assume that l=0 corresponds to the healthy condition $\left(A^{0},B^{0}\right)$ while any other l≥1 corresponds to a faulty condition. In addition, X,U,D,V are defined as the bounded polyhedral constraint sets [30,31]: $X=\left\{x\in R^{n}:H_{x}x\leq b_{x}\right\},U=\left\{u\in R^{n_{u}}:H_{u}u\leq b_{u}\right\},D=\left\{d\in R^{n_{d}}:H_{d}d\leq b_{d}\right\},V=\left\{v\in R^{b_{v}}:H_{v}v\leq b_{v}\right\}$, where $H_{x},H_{u},H_{d},H_{v},b_{x},b_{u},b_{d},b_{v}$ are predetermined.

**Remark** **1.** *The model (1) can represent some uncertainties. Firstly, the changes in the configuration of the system (i.e., $l\in [0,n_{f}]$) due to the appearance or disappearance of faults are essentially a description of the uncertainty of the system [1]. Secondly, the disturbance terms $\left(d_{k},\;v_{k}\right)$ included in the model can directly reflect the multiple uncertainties in the system. For instance, let $A^{l}=A^{0}$, $d_{k}=\Delta_{A}x_{k}+\delta_{k}$ with unknown but bounded term $\Delta_{A}$, (1) can represent a class of additive parametric uncertainty models; let $A^{l}=A^{0}$, $d_{k}=A^{0}\left(I-\Delta_{A}\right)x_{k}+\delta_{k}$, (1) can represent a class of multiplicative parametric uncertainty models; let $d_{k}$ be a time-varying/time-invariant uncertainty term only, (1) can represent the uncertainty case for a class of mechanistic models with bounded offsets of modeling error, etc. All of these scenarios can be used to reflect a mismatch between the model and the reality.*

**Remark** **2.** *The model (1) can represent both component and actuator faults [1]. For example, $A^{l}=A^{0}+\sum_{i=1}^{n}A^{i}\theta_{k}^{i}$ with unknown faulty factor $\theta_{k}^{i}$ can represent some component/parameter faults; $B^{l}=B^{0}diag\left\{\theta_{k}^{1},\theta_{k}^{2},\cdots ,\theta_{k}^{n_{u}}\right\}$ with $\theta_{k}^{i}\in \left[0,1\right]$ can describe some actuator effectiveness loss faults.*

For the sake of simplicity, the dynamics of the *l*-th system configuration can be rewritten as

(2) $$\begin{array}{cc} x_{k+1}^{l} & =A^{l}x_{k}^{l}+B^{l}u_{k}^{l}+d_{k},\;y_{k}^{l}=Cx_{k}^{l}+v_{k}\\ s.t.\;\; & x_{k}\in X,\;u_{k}\in U,\;d_{k}\in D,\;v_{k}\in V \end{array}$$

The following assumptions are given for systems (1) and (2).

**Assumption** **A1.** *The typical system configurations of concern can be modeled in advance, and these system configurations are controllable.*

**Remark** **3.** *We recognize that not all systems and faults can be tolerant by only one FTC method. Therefore, we make the above assumptions to explain the situations in which the proposed method can be applied.*

**Definition** **1.** *Let S be a neighborhood of the origin. The closed-loop trajectory of (1) is said to be Uniformly Ultimately Bounded (UUB) in S, if ∀ $x_{0}$, $\exists \;T(x_{0})>0$ such that $x_{k}\in S$ for $k\geq T(x_{0})$.*

The control objective is to construct an AFD-based robust and feasible AFTC strategy such that the states of the controlled system (1) can be steered inside a neighborhood of origin (i.e., UUB) in a way of minimizing the following optimization problem

(3) $$\begin{array}{cc} \min_{u_{k}}\; & J_{x_{k},u_{k}}=\sum_{t=0}^{\infty }U\left(x_{k+t},\;u_{k+t}\right)\\ s.t.\; & x_{k+1}^{l}=A^{l}x_{k}^{l}+B^{l}u_{k}^{l}+d_{k},\;y_{k}^{l}=Cx_{k}^{l}+v_{k}\\ \; & x_{k}\in X,\;u_{k}\in U,\;d_{k}\in D,\;v_{k}\in V\\ \; & l\in [0,\;n_{f}] \end{array}$$

where $U\left(x_{k},\;u_{k}\right)=x_{k}^{T}\Xi x_{k}+u_{k}^{T}\Theta u_{k}$, Ξ>0, Θ>0 is a utility function.

## 3. Main Results

### 3.1. The Overall Scheme of the Proposed AFD-Based Interpolation AFTC Method

**Figure 1:**
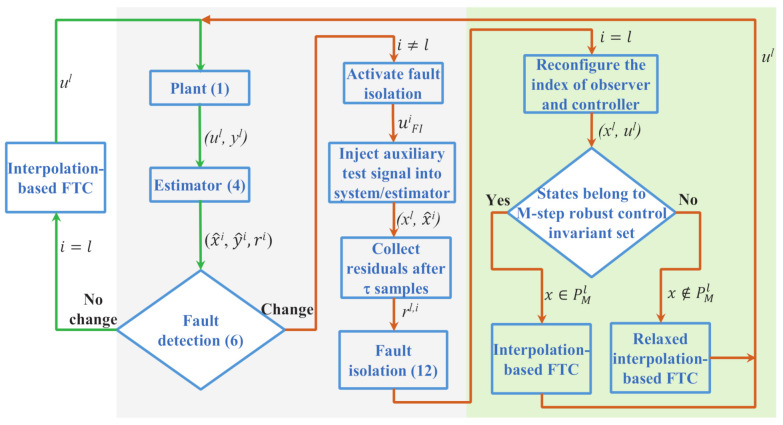
Scheme of AFD-based interpolation AFTC. The fault detection and active fault isolation constitute AFD, which will be designed in Section 3.2; AFTC consists of outer FTC, inner FTC and interpolation optimization, where outer FTC and interpolation optimization are designed in Section 3.4 and inner FTC with observer is designed in Section 3.3.

The overall scheme of the proposed AFD-based interpolation AFTC method is shown in Figure 1. In the subsequent analysis, we let that the index $l\in [0,n_{f}]$ denotes the unknown actual system operating condition and the index $i\in [0,n_{f}]$ denotes the recently identified system operating condition. Then, according to the flowchart in Figure 1, the AFTC method works as explained below. First, the I/O data of the practical system (i.e., the *l*th model) is collected by the *i*th estimator to give the state estimates ${\hat{x}}_{k}^{i}$ and generate the residuals $r_{k}^{i}$. Second, the fault detection unit performs change detection based on the estimator outputs. When there is no change (i.e., l=i), the interpolation control algorithm currently in use continues to regulate the system. When a change/fault is detected (i.e., l≠i), the fault isolation unit is activated, and in this case the pre-designed auxiliary test signal $u_{FI}^{i}$ is injected into the system and the estimator to perform modal discrimination. Next, after the practical system condition is isolated (i.e., i=l), the decision results of the fault isolation unit will update the operating condition index of the estimator and the reconfiguration controller. Next, the suitable interpolation optimization should be selected according to the location of states in relation to the feasible set of controller (i.e., robust control invariant set). Namely, if the states belong to the feasible set of the isolated controller, the general interpolation control is applied; otherwise, the relaxed interpolation control should be activated. Finally, these control actions will adjust the system states to the desired operating region. The design of each unit in this flowchart is given in detail below.

### 3.2. AFD: Fault/Mode Change Detection and Isolation

Without loss of generality, the following *i*-th observer is adopted to estimate states and generate residuals

(4) $$\begin{array}{cc} {\hat{x}}_{k+1}^{i} & =\left(A^{i}+L^{i}C\right){\hat{x}}_{k}^{i}+B^{i}u_{k}^{i}-L^{i}y_{k}^{i}\\ {\hat{y}}_{k}^{i} & =C{\hat{x}}_{k}^{i},r_{k}^{i}=y_{k}^{i}-{\hat{y}}_{k}^{i} \end{array}$$

where ${\hat{x}}_{k}^{i}\in R^{n}$ denotes the estimated state vector; ${\hat{y}}_{k}^{i}\in R^{n_{y}}$ is the estimated output vector; $r_{k}^{i}\in R^{n_{y}}$ is the generated residual signal that is used to provide key information of abnormal condition for achieving AFD. $L^{i}$ is the observer gain.

**Assumption** **2.** *For the sake of discussion, we assume that the observer (4) for each $i\in [0,\;n_{f}]$ has been designed in advance, and $\left(A^{i}+L^{i}C\right)$ is Schur stable. The detailed design conditions of $L^{i}$ are given in Theorem 1.*

**Remark** **4.** *In a cycle of AFD, the FI is always triggered by the FD [22]. Moreover, when a fault is detected at time $k_{d}$, the closed-loop FTC controller that is currently being used should preferably be put on standby to avoid that the feedback function hides the effect of the fault. In this setting, only the auxiliary input is used to stimulate the faulty system. In principle, the design of such an auxiliary input should (1) minimize the harmful influence to the currently matched system operation and (2) accurately identify and isolate the real system operating condition l.*

In Figure 1, there are two cases about the generated residual signal $r_{k}^{i}$. One is that the *i*th observer currently in use is matched to the real system mode *l*, and the other is the opposite. In the sequel, we will discuss the characteristics of the corresponding residuals for each of these two cases.

*(1)**Case I**(design of FD logic for i=l)*: First, based on Remark 4 and (1)–(4), the following estimation error system can be established:

(5) $$\begin{array}{c} e_{x^{i},k+1}\;=\;\left(A^{i}+L^{i}C\right)e_{x^{i},k}\;+\;L^{i}v_{k}\;+\;d_{k},\;r_{k}^{i}\;=\;Ce_{x^{i},k}\;+\;v_{k} \end{array}$$

where $e_{x^{i},k}=x_{k}^{i}-{\hat{x}}_{k}^{i}$. Given $i\in [0,n_{f}]$, the relevant disturbance term $L^{i}v_{k}+d_{k}$ is bounded by a deterministic set $\Delta_{e}^{i,i}=\left(L^{i}V\right)⊕D$. Then, based on a series of finite set iterations along (5) using $\Delta_{e}^{i,i}$, an approximate maximal RPI set $\Omega_{e}^{i,i}$ (see Definition 2) can be computed and the limit set of residual $r^{i}$ can be directly obtained as $R_{FD}^{i,i}=C\Omega_{e}^{i,i}⊕V$. According to Figure 1 and Remark 4, the detection of mode changes and the triggered action can be formulated as

(6) $$\left\{\begin{array}{cc} \; & r_{k}^{i}\notin R_{FD}^{i,i}\Rightarrow \mathrm{Mode}\;\mathrm{change}\;\Rightarrow \;\mathrm{Activate}\;\mathrm{FI}\\ \; & r_{k}^{i}\in R_{FD}^{i,i}\Rightarrow \mathrm{No}\;\mathrm{change}\;\;\;\;\;\Rightarrow \;\mathrm{Continue}\;\mathrm{detection}\; \end{array}\right.$$

**Remark** **5.** *Considering the possibility of fault occurrence, transformation, or recovery, we uniformly use mode change in (6) to indicate any phenomenon that causes a change in the system behavior.*

*(2)**Case II**(design of FI logic for i≠l)*: The case i≠l implies that the real status of system has changed and it generally leads to $r_{k}^{i}\notin R_{FD}^{i,i}$. In this case, the fault/mode isolation should be activated. According to the analysis method in [27] and Remark 4, an auxiliary input $u_{FI}^{i}$ will be used to replace the AFTC input $u_{C,k}^{i}$. A relevant augmentation representation is firstly constructed as

(7) $$\begin{array}{c} \chi_{k+1}^{l,i}\;=\;A_{\chi }^{l,i}\chi_{k}^{l,i}\;+\;B_{\chi }^{l,i}u_{FI}^{i}\;+\;E_{\chi }^{i}\sigma_{k},r_{k}^{l,i}\;=\;C_{\chi }\chi_{k}^{l,i}\;+\;D_{\chi }\sigma_{k} \end{array}$$

where $\chi_{k}^{l,i}={\left[\begin{array}{cc} {\left(x_{k}^{l}\right)}^{T} & {\left({\hat{x}}_{k}^{i}\right)}^{T} \end{array}\right]}^{T}$, $A_{\chi }^{l,i}=\left[\begin{array}{cc} A^{l} & 0\\ -L^{i}C & A^{i}+L^{i}C \end{array}\right]$, $\sigma_{k}\in E=\left\{{\left[\begin{array}{cc} d_{k}^{T} & v_{k}^{T} \end{array}\right]}^{T}:d\in D,v\in V\right\}$, $B_{\chi }^{l,i}=\left[\begin{array}{c} B^{l}\\ B^{i} \end{array}\right]$, $E_{\chi }^{i}=\left[\begin{array}{cc} I & 0\\ 0 & -L^{i} \end{array}\right]$, $C_{\chi }=\left[\begin{array}{cc} C & -C \end{array}\right]$, $D_{\chi }=\left[\begin{array}{cc} 0 & I \end{array}\right]$. Clearly, the term $E_{\chi }^{i}\sigma_{k}$ lies in the set $\Delta_{\chi }^{i}=E_{\chi }^{i}E$. Then, given $u_{FI}^{i}$ and based on Assumption 1, an approximate maximal RPI set $\Omega_{\chi }^{l,i}$ for each pair (l,i), i≠l, can be determined by finite set iterations along (7). Accordingly, the limit set that is used to achieve modal isolation can be obtained as $R_{FI}^{l,i}=C_{\chi }\Omega_{\chi }^{l,i}⊕D_{\chi }E$. The approximated calculation method of $R_{FI}^{l,i}$ is given in Appendix A.

A crucial condition for the existence of $u_{FI}^{i}$ that discriminates between configurations ζ and η in finite time is $R_{FI}^{\zeta ,i}\cap R_{FI}^{\eta ,i}=\emptyset$, ζ≠η. According to [27], such discrimination can be achieved by checking whether the distance between the two sets is positive. Without loss of generality, the following distance metric is defined as

(8) $$dis_{\zeta ,\eta }^{i}=\underset{\left(q_{\zeta }\in R_{FI}^{\zeta ,i},p_{\eta }\in R_{FI}^{\zeta ,i}\right)}{\inf}\;{\left\|q_{\zeta }-p_{\eta }\right\|}_{2}$$

Clearly, for each pair $\left(\zeta ,\;\eta \right)\in \{\left[0,n_{f}\right]∼i\},\zeta \neq \eta$, we need to solve (8) to determine a suitable auxiliary input $u_{FI}^{i}$ such that the distance metric $dis_{\zeta ,\eta }^{i}$ is positive. The distance metric (8) has the following properties.

**Lemma** **1.** *[27] The distance metric function $dis_{\zeta ,\eta }^{i}$ is convex and hence its maximum is reached on certain vertices of the input constraint set.*

Based on Remark 4 and Lemma 1, the optimization design problem of auxiliary input signal $u_{FI}^{i}$, $\forall i\in [0,n_{f}]$ can then be formulated as

(9) $$\left\{\begin{array}{cc} \;\; & \min\;\;\gamma \\ s.t.\; & dis_{\zeta ,\eta }^{i}>0;\;u_{FI}^{i}\in vert\left(\gamma U\right);\;\sigma_{k}\in E;\\ \; & \zeta ,\;\eta \in \{\left[0,n_{f}\right]∼i\};\;\zeta \neq \eta . \end{array}\right.$$

Once the problem in (9) is solved for each *i*, the corresponding separation hyperplane (denoted as $\Pi_{\zeta ,\eta }^{i}$) that is used to isolate the new mode can be further calculated through

(10) $$\begin{array}{cc} \Pi_{\zeta ,\eta }^{i} & =\{r:{\left(r-{\overset{˘}{r}}^{\eta }\right)}^{T}\left(r-{\overset{˘}{r}}^{\eta }\right)={\left(r-{\overset{˘}{r}}^{\zeta }\right)}^{T}\left(r-{\overset{˘}{r}}^{\zeta }\right)\}\\ \; & =\{r:{\left({\overset{˘}{r}}^{\zeta }-{\overset{˘}{r}}^{\eta }\right)}^{T}r=\frac{{\left({\overset{˘}{r}}^{\zeta }-{\overset{˘}{r}}^{\eta }\right)}^{T}\left({\overset{˘}{r}}^{\zeta }+{\overset{˘}{r}}^{\eta }\right)}{2}\} \end{array}$$

where ${\overset{˘}{r}}^{\zeta }\in R_{FI}^{\zeta ,i}$ and ${\overset{˘}{r}}^{\eta }\in R_{FI}^{\eta ,i}$ are two points at minimum distance from $\Pi_{\zeta ,\eta }^{i}$, and they can be determined when solving (9). Then, these off-line designed separation hyperplanes will be used for real-time isolation. For simplicity, the isolation function is constructed as

(11) $$Iso_{\zeta ,\eta }^{i}=sign\left[{\left({\overset{˘}{r}}^{\zeta }-{\overset{˘}{r}}^{\eta }\right)}^{T}r_{k}-\frac{{\left({\overset{˘}{r}}^{\zeta }-{\overset{˘}{r}}^{\eta }\right)}^{T}\left({\overset{˘}{r}}^{\zeta }+{\overset{˘}{r}}^{\eta }\right)}{2}\right]$$

Then, for the residual signals generated in real time, the online FI logic can be designed as

(12) $$\left\{\begin{array}{cc} \; & Iso_{\zeta ,\eta }^{i}>0\Rightarrow \mathrm{Mode}\;\zeta \;\mathrm{is}\;\mathrm{effective}\\ \; & Iso_{\zeta ,\eta }^{i}<0\Rightarrow \mathrm{Mode}\;\eta \;\mathrm{is}\;\mathrm{effective} \end{array}\right.$$

The current system mode can thus be discerned by making no more than $n_{f}$ comparisons using (11) and (12).

### 3.3. Integrated Design of Observer and Unconstrained Controller

When the practical system mode index $l\in [0,n_{f}]$ is isolated, the control reconfiguration should be activated immediately, i.e., the control action $u_{C,k}^{l}$ is reconfigured with the new isolated mode index *l*. Now we will design the control policy $u_{C,k}^{l}$. Here, we consider for now the case where the constraints (x∈X,u∈U) are not triggered and $u_{C,k}^{l}$ can then be designed only as an estimator-based robust feedback control policy $u_{C,k}^{l}=K^{l}{\hat{x}}_{k}^{l},\;\forall l\in \left[0,n_{f}\right]$. Under such settings, the closed-loop system dynamics can be obtained as

(13) $$\begin{array}{cc} x_{k+1}^{l} & =A^{l}x_{k}^{l}+B^{l}K^{l}{\hat{x}}_{k}^{l}+d_{k}\\ \; & =\left(A^{l}+B^{l}K^{l}\right)x_{k}^{l}-B^{l}K^{l}e_{x^{l},k}+d_{k}\\ \; & ={\bar{A}}^{l}x_{k}^{l}+{\bar{B}}^{l}e_{x^{l},k}+d_{k} \end{array}$$

where ${\bar{A}}^{l}=A^{l}+B^{l}K^{l}$, and ${\bar{B}}^{l}=-B^{l}K^{l}$.

On the other hand, by defining a virtual output variable vector $z_{k}^{l}=\left[\begin{array}{c} \Xi^{1/2}\\ 0 \end{array}\right]x_{k}^{l}+\left[\begin{array}{c} 0\\ \Theta^{1/2} \end{array}\right]u_{k}^{l}$, the unity function of cost function (3) can be represented by $U\left(x_{k}^{l},u_{k}^{l}\right)={\left(z_{k}^{l}\right)}^{T}z_{k}^{l}$. Then, the closed-loop virtual output by $u_{C,k}^{l}$ can be deduced as

(14) $$z_{k}^{l}={\bar{C}}^{l}x_{k}^{l}+{\bar{D}}^{l}e_{x^{l},k}$$

where ${\bar{C}}^{l}=\left[\begin{array}{c} \Xi^{1/2}\\ 0 \end{array}\right]+\left[\begin{array}{c} 0\\ \Theta^{1/2} \end{array}\right]K^{l}$ and ${\bar{D}}^{l}=-\left[\begin{array}{c} 0\\ \Theta^{1/2} \end{array}\right]K^{l}$.

According to [20,32], there may exist robustness interaction influences between estimation accuracy and unconstrained control performance, since the estimation error $e_{x^{l},k}$ disturbs the closed-loop system (13) and (14) whilst the unmodeled dynamics $d_{k}$ usually containing states can affect the estimation system (5). Hence, an integrated design of composite closed-loop system (5), (13) and (14) must be adopted to obtain the satisfactory observer gain $L^{l}$ and control gain $K^{l}$, $\forall l\in [0,n_{f}]$. The following composite closed-loop system is firstly established:

(15) $$\begin{array}{cc} \psi_{k+1}^{l} & ={\tilde{A}}^{l}\psi_{k}^{l}+{\tilde{B}}^{l}\varrho_{k}\\ z_{k}^{l} & ={\tilde{C}}^{l}\psi_{k}^{l} \end{array}$$

where $\psi_{k}^{l}={\left[\begin{array}{cc} e_{x^{l},k}^{T} & {\left(x_{k}^{l}\right)}^{T} \end{array}\right]}^{T}$, $\varrho_{k}={\left[\begin{array}{cc} v_{k}^{T} & d_{k}^{T} \end{array}\right]}^{T}$, ${\tilde{A}}^{l}=\left[\begin{array}{cc} {\tilde{A}}_{11}^{l} & 0\\ {\tilde{A}}_{21}^{l} & {\tilde{A}}_{22}^{l} \end{array}\right]$, ${\tilde{B}}^{l}=\left[\begin{array}{c} {\tilde{B}}_{1}^{l}\\ {\tilde{B}}_{2}^{l} \end{array}\right]$, ${\tilde{A}}_{11}^{l}=A^{l}+L^{l}C$, ${\tilde{A}}_{21}^{l}=-B^{l}K^{l}$, ${\tilde{A}}_{22}^{l}=A^{l}+B^{l}K^{l}$, ${\tilde{B}}_{1}^{l}=\left[\begin{array}{cc} L^{l} & I \end{array}\right]$, ${\tilde{B}}_{2}=\left[\begin{array}{cc} 0 & I \end{array}\right]$, and ${\tilde{C}}^{l}=\left[\begin{array}{cc} {\bar{D}}^{l} & {\bar{C}}^{l} \end{array}\right]$.

The following theorem presents the integrated design conditions of observer gain and unconstrained feedback gain.

**Theorem** **1.** *For each $l\in [0,n_{f}]$, a robust observer (5) and associated robust feedback control policy $u_{C,k}=K^{l}{\hat{x}}_{k}^{l}$ can be integratedly determined, if some decision variables α>0, β>0, $P_{1}^{l}={\left(P_{1}^{l}\right)}^{T}>0$, $P_{2}^{l}={\left(P_{2}^{l}\right)}^{T}>0$, $Y_{1}^{l}$, $Y_{2}^{l}$, ${\bar{K}}^{l}$, ${\bar{L}}^{l}$ exist as the solutions to the following optimization problem:*

$$\begin{array}{cc} \; & \min_{P_{1}^{l},P_{2}^{l},Y_{1}^{l},Y_{2}^{l},{\bar{K}}^{l},{\bar{L}}^{l}}\varsigma \alpha +\left(1-\varsigma \right)\beta \end{array}$$

(16a) $$\begin{array}{cc} s.t.\; & \left[\begin{array}{ccc} I-P_{1}^{l} & ⋄ & ⋄\\ 0 & -\alpha^{2}I & ⋄\\ \Gamma_{31l} & \Gamma_{32l} & P_{1}^{l}-Y_{1}^{l}-{\left(Y_{1}^{l}\right)}^{T} \end{array}\right]<0 \end{array}$$

(16b) $$\begin{array}{cc} \; & \left[\begin{array}{ccccc} P_{2}^{l}\;-\;Sym\left(Y_{2}^{l}\right) & ⋄ & ⋄ & ⋄ & ⋄\\ 0 & P_{2}\;-\;Sym\left(Y_{2}^{l}\right) & ⋄ & ⋄ & ⋄\\ 0 & 0 & -\beta^{2}I & ⋄ & ⋄\\ \Upsilon_{41l} & \Upsilon_{42l} & {\tilde{B}}_{2} & -P_{2}^{l} & ⋄\\ \Upsilon_{51l} & \Upsilon_{52l} & 0 & 0 & -I \end{array}\right]\;\;\;<\;\;0 \end{array}$$

*where $Sym\left(Y_{2}^{l}\right)=Y_{2}^{l}+{\left(Y_{2}^{l}\right)}^{T}$, $\Gamma_{31l}=Y_{1}^{l}A^{l}+{\bar{L}}^{l}C$, $\Gamma_{32l}=\left[\begin{array}{cc} {\bar{L}}^{l} & Y_{1}^{l} \end{array}\right]$, $\Upsilon_{41l}=-B^{l}{\bar{K}}^{l}$, $\Upsilon_{42l}=A^{l}{\left(Y_{2}^{l}\right)}^{T}+B^{l}{\bar{K}}^{l}$, ${\tilde{B}}_{2}^{l}=\left[\begin{array}{cc} 0 & I \end{array}\right]$, $\Upsilon_{51l}=-\left[\begin{array}{c} 0\\ \Theta^{1/2} \end{array}\right]{\bar{K}}^{l}$, $\Upsilon_{52l}=\left[\begin{array}{c} \Xi^{1/2}\\ 0 \end{array}\right]{\left(Y_{2}^{l}\right)}^{T}+\left[\begin{array}{c} 0\\ \Theta^{1/2} \end{array}\right]{\bar{K}}^{l}$, ς∈(0,1). Once the above optimization is solved, the parameters of observer and feedback gain can be calculated by $K^{l}={\bar{K}}^{l}{\left({\left(Y_{2}^{l}\right)}^{T}\right)}^{-1}$ and $L^{l}={\left(Y_{1}^{l}\right)}^{-1}{\bar{L}}^{l}$, respectively.*

**Proof.**  The proof of Theorem 1 is given in Appendix B.    □

### 3.4. Constrained AFTC: Reconfigured Interpolating Control

Based on the set-theoretic concepts in [28,31], several invariant sets are defined.

**Definition** **2.** *Given the controller $u_{C,k}^{l}=K^{l}{\hat{x}}_{k}^{l}$, the set $\Omega_{RPI}^{l}\subseteq X$ is a robust positive invariant set (RPI-set) for closed-loop system (13) subject to constraint $x_{k}^{l}\in X$ if for any $x_{0}^{l}\in \Omega_{RPI}^{l}$ we have $x_{k}^{l}\in \Omega_{RPI}^{l}$ for all ${\bar{B}}^{l}e_{x^{l},k}+d_{k}$, k>0. Moreover, $\Omega_{MRPI}^{l}$ is the maximal RPI-set if $\Omega_{MRPI}^{l}$ contains all the RPI-sets of constrained closed-loop system (13) in X. For simplicity, $\Omega_{MRPI}^{l}$ is represented in the polyhedral form of $\Omega_{MRPI}^{l}=\left\{x^{l}:F_{I}^{l}x^{l}\leq g_{I}^{l}\right\}$.*

The following enlarged invariant set is further defined for some constrained allowable control inputs.

**Definition** **3.** *Given the lth model of (2) and the constraints (X,U), the set $\Omega_{RCI}^{l}\subseteq X$ is a robust control invariant set (RCI-set), if for any $x_{0}^{l}\in \Omega_{RCI}$ there exists an admissible control input $u_{k}^{l}\in U$ such that all the state updates satisfy $x_{k}^{l}\in \Omega_{RCI}$ for all $d_{k}$ and $e_{x^{l},k}$, k>0. Similarly, the maximal RCI-set $\Omega_{MRCI}$ contains all robust RCI-sets.*

Generally, the determination of $\Omega_{MRCI}$ is computationally demanding, in particular for high-dimension systems. As an alternative, the *M*-step robust control invariant set can be used.

**Definition** **4.** *The set $P_{M}^{l}\subseteq X$ is defined as a M-step robust control invariant set for the lth model of (2) with respect to the constraints (X,U), if there exists an admissible control sequence such that all states $x_{k}^{l}\in P_{M}^{l}$ can be steered into $\Omega_{MRPI}^{l}$ in no more than M steps. For simplicity, $P_{M}^{l}$ is described as $P_{M}^{l}=\left\{x^{l}:F_{M}^{l}x^{l}\leq g_{M}^{l}\right\}$.*

In general, two cases exist for the location of the states of system after the active FI is completed, namely $x_{k}^{l}\in P_{M}^{l}$ and $x_{k}^{l}\notin P_{M}^{l}$. In the sequel, we will construct an interpolating FTC strategy for each of these two cases.

*(1)**Case I**($x_{k}^{l}\in P_{M}^{l}$ after FI)*: Firstly, in order to get $\Omega_{MRPI}^{l}$ of (13), the bounded set of ${\bar{B}}^{l}e_{x^{l},k}+d_{k}$ should be determined. By a series of finite set iterations along (5), the disturbance invariant set of $e_{x^{l}}$ subject to $v_{k}\in V$ and $d_{k}\in D$ has been computed as $\Omega_{e}^{l,l}$. Then, we have ${\bar{B}}^{l}e_{x^{l},k}+d_{k}\in \left({\bar{B}}^{l}\Omega_{e}^{l,l}⊕D\right)$. Further, the *Procedure 2.1* in [28] can be referred to calculate $\Omega_{MRPI}^{l}$ of (13).

In order to describe the control actions that can regulate the state $x_{k}^{l}$ from $P_{M}^{l}$ back to $\Omega_{MRPI}^{l}$ in no more than *M* steps, an augmented control sequence $U_{M,k}^{l}={\left[\begin{array}{cccc} {\left(u_{IC,k}^{l}\right)}^{T} & {\left(u_{IC,k+1}^{l}\right)}^{T} & \cdots & {\left(u_{IC,k+M-1}^{l}\right)}^{T} \end{array}\right]}^{T}$ is defined. In fact, these actions are expected to regulate the dynamic behavior of the system in the following manner:

(17) $$\begin{array}{cc} \; & x_{k+1}^{l}=A^{l}x_{k}^{l}+B^{l}u_{IC,k}^{l}+d_{k}\in P_{M}^{l}\\ \; & \;\;\vdots \\ \; & x_{k+M-1}^{l}\;=\;A^{l}x_{k\;+\;M\;-\;2}^{l}\;+\;B^{l}u_{IC,k\;+\;M-2}^{l}\;+\;d_{k\;+\;M-\;2}\;\in \;P_{M}^{l}\\ \; & x_{k+M}^{l}\;=\;A^{l}x_{k\;+\;M\;-\;1}^{l}\;+\;B^{l}u_{IC,k\;+\;M-\;1}^{l}\;+\;d_{k\;+\;M\;-\;1}\;\in \;\Omega_{MRPI}^{l} \end{array}$$

Obviously, in (17) we can observe that the migration process of states can be approximately deduced by the current initial state $x_{k}^{l}$ and a sequence of inputs $U_{M,k}^{l}$. Considering the constraints with Definition 4, we can further describe the maximal admissible control domain of the system (1) with respect to the corresponding control inputs in terms of the following half-space representation for the augmented state space $Q_{M}^{l}=\left\{x^{l},U_{M}^{l}\right\}$:

(18) $$Q_{M}^{l}=\left\{x^{l},U_{M}^{l}:{\bar{F}}_{M}^{l}\left[\begin{array}{c} x^{l}\\ U_{M}^{l} \end{array}\right]\leq {\bar{g}}_{M}^{l}\right\}$$

**Remark** **6.** *Given the previously obtained $\Omega_{MRPI}^{l}$ and certain M, the augmented set $Q_{M}^{l}$ can be calculated by following the algorithm in [28]. In addition, by comparing the definition in (18) and Definition 4, it can be seen that $P_{M}^{l}$ is a projection of $Q_{M}^{l}$ onto the state space.*

Without loss of generality, any state vector $x_{k}^{l}\in P_{M}^{l}$ can be decomposed as a convex combination form

(19) $$x_{k}^{l}=s_{k}^{l}x_{O,k}^{l}+\left(1-s_{k}^{l}\right)x_{I,k}^{l}$$

where $x_{I,k}^{l}\;\in \;\Omega_{MRPI}^{l}$ denotes an inner state vector while $x_{O,k}^{l}\;\in \;P_{M}^{l}\;∼\;\Omega_{MRPI}^{l}$ denotes an outer state vector. $s_{k}^{l}\;\in \;\left[0,1\right]$ is the so-called interpolation coefficient. Since $x_{I,k}$ has already inside $\Omega_{MRPI}^{l}$, the previously designed unconstrained optimal control law by $K^{l}x_{I,k}^{l}$ can be directly adopted to achieve UUB regulation of $x_{I,k}^{l}$ robustly. Thus, for $x_{k}^{l}\notin \Omega_{MRPI}^{l}$, (19) means that the problem of finding $U_{M,k}^{l}$ to regulate state $x_{k}^{l}$ back to $\Omega_{MRPI}^{l}$ can be transformed into the problem of solving $U_{M,k}^{l}$ to regulate state $x_{O,k}^{l}$ back into $\Omega_{MRPI}^{l}$.

In line with the above state decomposition (19), the following interpolated FTC strategy for the *l*th model is constructed

(20) $$u_{C,k}^{l}=s_{k}^{l}u_{IC,k}^{l}+\left(1-s_{k}^{l}\right)u_{I,k}^{l}$$

where $u_{I,k}^{l}=K^{l}x_{I,k}^{l}$ is the inner FTC law while $u_{IC,k}^{l}$ is the outer FTC law to be determined. It should be noted that $u_{I,k}^{l}$ is the optimal unconstrained terminal control law, and it generally presents high control performance. However, for $x_{O,k}^{l}\in P_{M}^{l}∼\Omega_{MPRI}^{l}$, the constraints will be activated and the performance might be poor. Thus, in order to make the high-performance inner controller as dominant as possible and minimize the constraint activation influence simultaneously, it is desirable to set $s_{k}^{l}$ as small as possible. This can be achieved by solving the following optimization problem:

(21) $$\left\{\begin{array}{cc} {\tilde{s}}_{k}^{l}= & \min_{s_{k}^{l},x_{I,k}^{l},x_{O,k}^{l},U_{M,k}^{l}}\;\;s_{k}^{l}\\ s.t.\; & F_{I}^{l}x_{I,k}^{l}\leq g_{I}^{l};\;{\tilde{F}}_{M}^{l}\left[\begin{array}{c} x_{O,k}^{l}\\ U_{M,k}^{l} \end{array}\right]\leq {\tilde{g}}_{M}^{l};\\ \; & x_{k}^{l}=s_{k}^{l}x_{O,k}^{l}+\left(1-s_{k}^{l}\right)x_{I,k}^{l};\;0\leq s_{k}^{l}\leq 1. \end{array}\right.$$

The first constraint in (21) is used to ensure $x_{I,k}^{l}\in \Omega_{MRPI}^{l}$; the second inequality is used to ensure that there exists $U_{M,k}^{l}$ such that $x_{O,k}^{l}\in {\tilde{P}}_{M}^{l}\subseteq P_{M}^{l}$ and $x_{O,k+M}^{l}\in \Omega_{MRPI}^{l}$; the third equation guarantees a smooth convex interpolation between $x_{I,k}^{l}$ and $x_{O,k}^{l}$ and also achieves a smooth interpolation between the associated two control laws.

**Remark** **7.** *In view of the influence of estimation error on the feasibility of optimization, we have contracted the constraint condition in (18), and obtained the second constraint condition in (21). Specifically, by setting $u_{IC,k}^{l}=K^{l}x_{O,k}^{l}+c_{k}^{l}$, there is $u_{C,k}^{l}=s_{k}^{l}u_{IC,k}^{l}+\left(1-s_{k}^{l}\right)u_{I,k}^{l}=s_{k}^{l}\left(K^{l}x_{O,k}^{l}+c_{k}^{l}\right)+\left(1-s_{k}^{l}\right)u_{I,k}^{l}=s_{k}^{l}K^{l}x_{O,k}^{l}+\left(1-s_{k}^{l}\right)K^{l}x_{I,k}^{l}+s_{k}^{l}c_{k}^{l}=K^{l}\left(s_{k}^{l}x_{O,k}^{l}+\left(1-s_{k}^{l}\right)x_{I,k}^{l}\right)+s_{k}^{l}c_{k}^{l}=K^{l}x_{k}^{l}+{\bar{c}}_{k}^{l}$, where ${\bar{c}}_{k}^{l}=s_{k}^{l}c_{k}^{l}$. Then, following the augmentation analysis technique in dual-mode predictive control [20], we can calculate a disturbance invariant set of $\left[x_{k};U_{M,k}^{l}\right]$ that is driven by $e_{x^{l},k}$ and $d_{k}$. Further, based on the constraint tightening, a conservative constraint set ${\tilde{Q}}_{M}^{l}=\left\{x^{l},U_{M}^{l}:{\tilde{F}}_{M}^{l}\left[\begin{array}{c} x^{l}\\ U_{M}^{l} \end{array}\right]\leq {\tilde{g}}_{M}^{l}\right\}$ can be determined, where ${\tilde{P}}_{M}^{l}$ is a projection of ${\tilde{Q}}_{M}^{l}$ onto the state space.*

Since $s_{k}^{l},\;x_{I,k}^{l},\;x_{O,k}^{l}$ are unknown, the optimization (21) is nonlinear. Let $b_{O,k}^{l}=s_{k}^{l}x_{O,k}^{l}$, $b_{I,k}^{l}=\left(1-s_{k}^{l}\right)x_{I,k}^{l}$, and $T_{M,k}^{l}=s_{k}^{l}U_{M,k}^{l}$, the above optimization problem (21) can be then simplified as a linear programming problem:

(22) $$\left\{\begin{array}{cc} {\tilde{s}}_{k}^{l}= & \min_{s_{k}^{l},b_{O,k}^{l},T_{M,k}^{l}}\;\;s_{k}^{l}\\ s.t.\; & F_{I}^{l}\left(x_{k}^{l}-b_{O,k}^{l}\right)\leq \left(1-s_{k}^{l}\right)g_{I}^{l};\\ \; & {\tilde{F}}_{M}^{l}\left[\begin{array}{c} b_{O,k}^{l}\\ T_{M,k}^{l} \end{array}\right]\leq s_{k}^{l}{\tilde{g}}_{M}^{l};\;0\leq s_{k}^{l}\leq 1. \end{array}\right.$$

When the optimal solution of (22) is obtained, the reconfigured interpolation FTC can then be constructed as $u_{C,k}^{l}=T_{M1,k}^{l}+K^{l}\left(x_{k}^{l}-b_{O,k}^{l}\right)$, where $T_{M1,k}^{l}$ is the first control input in $T_{M,k}^{l}$.

*(2)**Case II**($x_{k}^{l}\notin P_{M}^{l}$ after FI)*: The soft constraint methods are employed to ensure that states outside $P_{M}^{l}$ can also be steered into $\Omega_{MRPI}^{l}$ after the fault is isolated. Depending on the requirements of the actual system for state constraints and input constraints, there exist two general ways to design soft constraints [33,34]. The first is that the input constraints must not be violated while the boundaries of the state constraints can be relaxed appropriately. The other is that the boundaries of both constraints can be adjusted. In either case, the relaxation variable introduced by the soft constraints is non-zero only when the original constraints are violated. Once the original constraints are restored, the relaxation variable must be zero. For the sake of simplicity, the second strategy is adopted and we design the following soft constrained interpolating control algorithm. First of all, we suppose that the maximal admissible control domain (18) can be relaxed to contain states $x_{k}^{l}\notin P_{M}^{l}$ as follows:

(23) $${\tilde{Q}}_{\varsigma_{k},M}^{l}=\left\{x^{l},U_{M}^{l}:{\tilde{F}}_{M}^{l}\left[\begin{array}{c} x^{l}\\ U_{M}^{l} \end{array}\right]\leq {\tilde{g}}_{M}^{l}+\varsigma_{k}^{l}\Lambda \right\}$$

where $\varsigma_{k}^{l}\geq 0$ is the relaxation variable and Λ can be a column vector of ones or an arithmetic progression vector with the first term 1 and common difference −κ∈[−1,0]. Note that the soft constraints by (23) implicitly define an enlarged M−step robust control invariant set ${\tilde{P}}_{\varsigma_{k},M}^{l}$ for systems (1) with relaxed constraints of states and inputs.

Then, in a similar way to formulate (19) and (20), we can also update the interpolations of states and inputs for $x_{I,k}^{l}\in \Omega_{MRPI}^{l}$ and $x_{O,k}^{l}\in {\tilde{P}}_{\varsigma_{k},M}^{l}∼\Omega_{MRPI}^{l}$. Slightly different from the optimization objective of (21), here the slack variable $\varsigma_{k}$ also needs to be minimized, i.e., the degree of constraint violation of ${\tilde{P}}_{M}^{l}$ should be minimized. To this point, we can further establish the following optimization problem through the same design of variables as (22):

(24) $$\left\{\begin{array}{cc} {\tilde{\mu }}_{k}^{l}= & \min_{s_{k}^{l},b_{O,k}^{l},T_{M,k}^{l},{\bar{\varsigma }}_{k}^{l}}\;\;\varepsilon_{1}{\bar{\varsigma }}_{k}^{l}+\varepsilon_{2}s_{k}^{l}\\ s.t.\; & F_{I}^{l}\left(x_{k}^{l}-b_{O,k}^{l}\right)\leq \left(1-s_{k}^{l}\right)g_{I}^{l};\;0\leq s_{k}^{l}\leq 1;\\ \; & {\tilde{F}}_{M}^{l}\left[\begin{array}{c} b_{O,k}^{l}\\ T_{M,k}^{l} \end{array}\right]\leq s_{k}^{l}{\tilde{g}}_{M}^{l}+{\bar{\varsigma }}_{k}^{l}\Lambda ;\;{\bar{\varsigma }}_{k}^{l}=s_{k}^{l}\varsigma_{k}^{l}. \end{array}\right.$$

where $\varepsilon_{1}+\varepsilon_{2}=1$. In order to highlight the function of soft constraint FTC, $\varepsilon_{1}$ is generally set to be larger than $\varepsilon_{2}$.

### 3.5. The AFD-Based Reconfigured Interpolation FTC Algorithm

A binary parameter $\varepsilon_{3}$ is introduced to unify the optimization problems of (22) and (24):

(25) $$\left\{\begin{array}{cc} {\tilde{\mu }}_{k}^{l}= & \min_{s_{k}^{l},b_{O,k}^{l},T_{M,k}^{l},{\bar{\varsigma }}_{k}^{l}}\;\;\varepsilon_{3}\left(\varepsilon_{1}{\bar{\varsigma }}_{k}^{l}+\varepsilon_{2}s_{k}^{l}\right)+\left(1-\varepsilon_{3}\right)s_{k}^{l}\\ s.t.\; & F_{I}^{l}\left(x_{k}^{l}-b_{O,k}^{l}\right)\leq \left(1-s_{k}^{l}\right)g_{I}^{l};\;0\leq s_{k}^{l}\leq 1;\\ \; & {\tilde{F}}_{M}^{l}\left[\begin{array}{c} b_{O,k}^{l}\\ T_{M,k}^{l} \end{array}\right]\leq s_{k}^{l}{\tilde{g}}_{M}^{l}+\varepsilon_{3}{\bar{\varsigma }}_{k}^{l}\Lambda ;\;0\leq {\bar{\varsigma }}_{k}^{l}. \end{array}\right.$$

By setting $\varepsilon_{3}=1$, (25) reduces to (24), which is used to achieve soft constrained interpolating control for the case $x_{k}^{l}\notin {\tilde{P}}_{M}^{l}$. By setting $\varepsilon_{3}=0$, (25) reduces to (22) and the standard interpolating control based FTC can then be achieved. All the above developments allow us to write down Algorithm 1.
**Algorithm 1** AFD-based interpolation AFTC.**Off-line Design:** Given system (1), objective (3) and sets (X,U,D,V). Complete the following designs: Solve Theorem 1 to obtain $K^{l},\;L^{l}$, $\forall l=0,1,\cdots ,n_{f}$; Based on (5), calculate the limit set of residual $R_{FD}^{i,i}$, $\forall i=0,1,\cdots ,n_{f}$; Solve the optimization problem (9) to obtain $u_{FI}^{i}$ and associated points ${\overset{˘}{r}}^{\zeta }$, ${\overset{˘}{r}}^{\eta }$, $\forall i,\zeta ,\eta =0,1,2,\cdots ,n_{f},i\neq \zeta$, i≠η, ζ≠η, and construct separation hyperplane (10); Formulate the FD logic (6) using $R_{FD}^{i,i}$ and construct the FI logic (12) using (11); Calculate $\Omega_{MRPI}^{l}$ according to Definition 2 and calculate ${\tilde{Q}}_{M}^{l}$ based on (18) and Remarks 6 and 7.**On-line Implementation:** Set ς, $\varepsilon_{1}$, $\varepsilon_{2}$, μ∈[0,1], state $x_{0}={\hat{x}}_{0}\in P_{M}^{0}$ and $u_{FI}^{0}=0$. Solve (25) with $\varepsilon_{3}=0$ to obtain $u_{C,0}^{0}\leftarrow T_{M1,0}^{0}+K^{0}\left({\hat{x}}_{0}-b_{O,0}^{0}\right)$. Let i←0, k←0$u_{0}\leftarrow u_{C,0}^{0}$ and perform the following actions:1:$\left({\hat{x}}_{k},r_{k}\right)\leftarrow$(4); ⊲ Using (4) to estimate $\left({\hat{x}}_{k},r_{k}\right)$2:**if**$r_{k}\in R_{FD}^{i,i}$**then**3: Solve (25) with $\varepsilon_{3}=0$ to compute $u_{C,k}^{i}\leftarrow T_{M1,k}^{i}+K^{i}\left({\hat{x}}_{k}-b_{O,k}^{i}\right)$; Set $u_{FI}^{i}\leftarrow 0$, $u_{k}\leftarrow u_{C,k}^{i}$, k←k+1, and go to step 1;4:**else**⊲ A fault is detected5: Set $u_{C,k}^{i}\leftarrow 0$, $u_{k}\leftarrow u_{FI}^{i}$;6:**end if**7:**do**8: $\left({\hat{x}}_{k+\tau },r_{k+\tau }\right)\leftarrow$(4); ⊲ Generate τ residuals9: Find ζ such that $Iso_{\zeta ,\eta }^{i}>0,\forall \eta \in \left[0,n_{f}\right]∼i,\zeta \neq \eta$;10:**end do**11:i←ζ, k←k+τ;12:**if**${\hat{x}}_{k}\in {\tilde{P}}_{M}^{i}$ then13: Go to step 3;14:**else**⊲${\hat{x}}_{k}\notin {\tilde{P}}_{M}^{i}$15: Solve (25) with $\varepsilon_{3}=1$ to compute $u_{C,k}^{i}\leftarrow T_{M1,k}^{i}+K^{i}\left({\hat{x}}_{k}-b_{O,k}^{i}\right)$; Set $u_{FI}^{i}\leftarrow 0$, $u_{k}\leftarrow u_{C,k}^{i}$, k←k+1, $\left({\hat{x}}_{k},r_{k}\right)\leftarrow$(4), and go to step 12;16:**end if**

## 4. Algorithm Verification by a Wastewater Treatment Plant Model

### 4.1. System Model and Parameters

The purpose of a wastewater treatment plant is to purify the sewage and return clean water to the river. Activated sludge process (ASP) is a very important part of the cleaning procedure [35]. Generally, ASP systems usually consist of a bioreactor and a settler. Bioreactors mainly rely on suspended microorganisms for biodegradation of dissolved substrate. After that, the suspended micro-organisms are completely separated in the settler. Some of the degraded biomass will be recycled to the bioreactor for further purification, while the remaining biomass will be discharged to maintain the balance of limited organisms in the ASP system. The energy needed for the reaction is provided by the dissolved oxygen, and the resulting carbon dioxide is in turn released. In [36], a simplified state-space error model describing the mass balances in ASP systems is built around the equilibrium point $\left(X_{P},U_{P}\right)=({\left[122.7342\;49.4714\;196.3750\;6.8300\right]}^{T},$[0.061.35]). Here, to achieve the fault tolerant mass balance of ASP systems, some uncertain parameters along the model in [36] are additionally considered as follows:

(26) $$\begin{array}{cc} \; & A\;=\;\;\;\left[\begin{array}{cccc} 0.7685\;\;-\;\;\Delta_{A} & 0.1551 & 0.0576 & 0.1273\\ -0.1438 & 0.4137\;\;+\;\;\Gamma_{A} & -0.0859 & -0.013\\ -0.0109 & -0.0175 & 0.0026\;\;+\;\;\Delta_{A} & -0.0018\\ 0.3396 & 0.0377 & 0.0253 & 0.8335\;\;-\;\;\Gamma_{A} \end{array}\right]\\ \; & B=\left[\begin{array}{cc} -250.0774 & 0.9268\\ 398.0189 & -1.4129\\ -13.8515 & 2.0454\\ 102.6287 & 0.18 \end{array}\right]\;\;\left[\begin{array}{cc} \Delta_{B} & 0\\ 0 & \Gamma_{B} \end{array}\right],\;C={\left[\begin{array}{cc} 0 & 0\\ 1 & 0\\ 0 & 1\\ 0 & 0 \end{array}\right]}^{T} \end{array}$$

We assume that two types of faults can appear: $\left(\Delta_{A}\;=\;0.2,\;\Delta_{B}\;=\;0.7,\;\;\Gamma_{A}\;=\;\Gamma_{B}\;=\;0\right)$ and $\left(\Delta_{A}\;=\Delta_{B}\;=\;\;0,\;\Gamma_{A}\;=\;0.3,\;\Gamma_{B}\;=\;0.6\right)$. The former is identified as fault l=1 (faulty mode 1) and the latter is identified as fault l=2 (faulty mode 2). Clearly, the health condition l=0 (healthy mode 0) is indicated when $\left(\Delta_{A}\;=\Gamma_{A}\;=\Delta_{B}\;=\Gamma_{B}\;=\;0\right)$. The other parameters are: $\Xi \;=\;0.1I_{4},\Theta \;=\;0.02I_{2},\;n_{f}\;=\;2,d_{k}\;\in \;\left[-0.51_{4}\;0.51_{4}\right],v_{k}\;\in \;\left[-0.5I_{2}\;0.5I_{2}\right]$, and

(27) $$\left[\begin{array}{c} -90.0342\\ -37.4714\\ -143.8750\\ -5.9300 \end{array}\right]\leq x_{k}\leq \left[\begin{array}{c} 137.2658\\ 15.5286\\ 13.6250\\ 73.1700 \end{array}\right],\;\left[\begin{array}{c} -0.0600\\ -1.3500 \end{array}\right]\leq u_{k}\leq \left[\begin{array}{c} 2.3400\\ 13.6500 \end{array}\right]$$

### 4.2. Offline Design of AFD and AFTC According to Algorithm 1 and Relevant Validation

According to Algorithm 1, the following parameters of AFTC policy are designed.

Firstly, by solving Theorem 1, the integrated parameters of observer $L^{l}$ and inner FTC gain matrix $K^{l}$ are obtained as

(28) $$\begin{array}{cc} \; & L^{0}=\left[\begin{array}{cc} 4.7216 & 0.2884\\ -1.2215 & -0.0229\\ -0.0518 & -0.0051\\ 9.4863 & 0.6130 \end{array}\right],\;K^{0}=\left[\begin{array}{cccc} -0.0016 & -0.0010 & 0.0002 & -0.0021\\ -0.8410 & -0.2855 & -0.0381 & -0.7295 \end{array}\right]\\ \; & L^{1}=\left[\begin{array}{cc} 0.1650 & -0.0290\\ -0.4078 & 0.0669\\ 0.0095 & -0.1675\\ 0.7874 & 0.0267 \end{array}\right],\;K^{1}=\left[\begin{array}{cccc} -0.0031 & -0.0017 & -0.0002 & -0.0048\\ -0.6755 & -0.2408 & -0.0910 & -0.8303 \end{array}\right]\\ \; & L^{2}=\left[\begin{array}{cc} 0.6487 & -0.0128\\ -0.7432 & 0.0605\\ 0.0036 & -0.0024\\ 0.6966 & 0.0089 \end{array}\right],\;K^{2}=\left[\begin{array}{cccc} -0.0014 & -0.0021 & 0.0001 & -0.0008\\ -0.5591 & -0.2930 & 0.0015 & -0.2738 \end{array}\right] \end{array}$$

Secondly, by using the disturbance set $\Delta_{e}^{i,i}$ for 3-step set iteration along (5), the limit sets of residual for each i=0,1,2 are approximately calculated, where the H-representations of $R_{FD}^{0,0}$, $R_{FD}^{1,1}$, and $R_{FD}^{2,2}$ have 23, 38, and 47 inequalities, respectively. Due to the page limit, they are not listed here.

**Figure 2:**
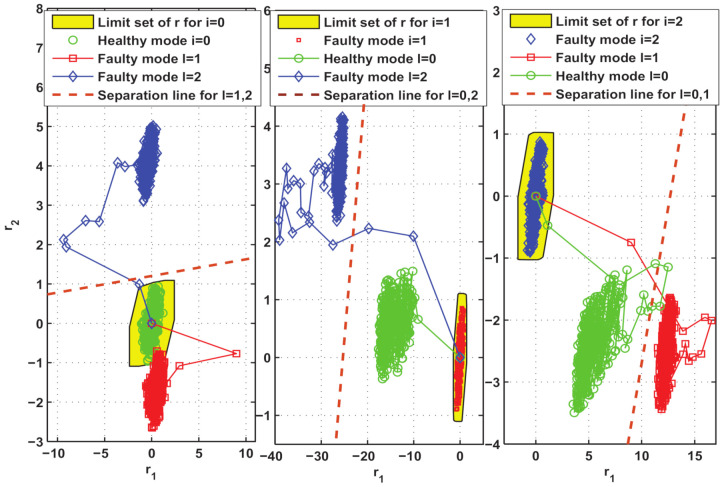
Test of the isolation effect of the constructed active fault isolation method in three scenarios.

Thirdly, by solving optimization problem (9), some suitable choices of test input signals are determined as $u_{FI}^{0}=1.5\times U.V\left(1\right)$, $u_{FI}^{1}=1.3\times U.V\left(1\right)$, $u_{FI}^{2}=1.1\times U.V\left(1\right)$, respectively. Here, U.V(1) is used to denote the first vertex of the V-representation of set U. In order to clearly describe the relationship between the FD limit set and the FI separation line, we simulated the residual responses by injecting the above test input signal excitation in different modes of the system. As shown in Figure 2, the AFD can be successfully achieved as long as the residual value exceeds the relevant separation line. Here, the isolation can be accomplished in a maximum of six steps.

**Figure 3:**
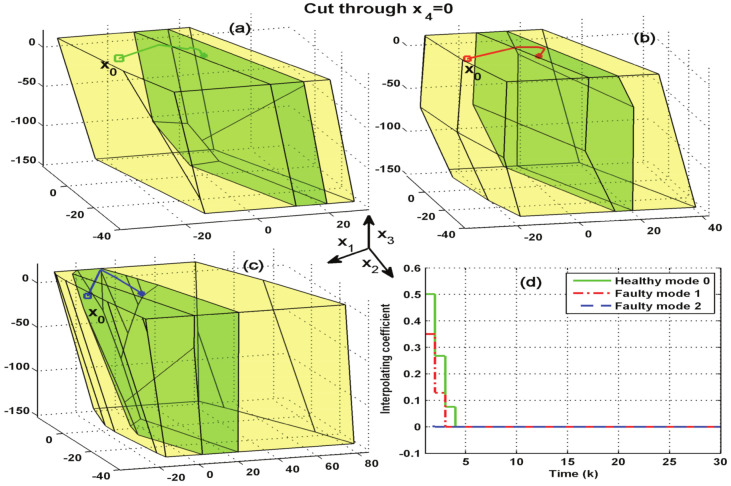
Test of the control effect of the developed interpolating AFTC. The yellow area represents the set $\Omega_{MRPI}^{l}$ and the green area represents the set $P_{M}^{l}$. (**a**) Invariant sets for health mode l=0; (**b**) invariant sets for fault mode l=1; (**c**) invariant sets for fault mode l=2; (**d**) interpolation coefficient ${\tilde{s}}_{k}$.

Next, the robust invariant sets $\Omega_{MRPI}^{l}$ and $P_{M}^{l}$ are calculated for l=0,1,2, respectively. In order to describe the relationship among the interpolating AFTC, the controlled states and the corresponding invariant set for each mode, the evolution of an arbitrary initial state $x_{0}={\left[-20\;10\;-10\;-1.83\right]}^{T}$ is simulated. The results of the first three states are shown in Figure 3. It can be seen from Figure 3a,b that $x_{0}$ belongs to $P_{M}^{l}∼\Omega_{MRPI}^{l}$, l=0,1. Therefore, as shown in sub-Figure 3d, the corresponding interpolation coefficients are not zero and $x_{0}$ is adjusted back to $\Omega_{MRPI}^{l}$ in 2-3 steps. Figure 3c illustrates that $x_{0}$ belongs to $\Omega_{MRPI}^{2}$. Hence, the associated interpolation coefficient in Figure 3d is zero.

### 4.3. Simulation Results and Analysis of the above Designed AFD-Based AFTC Method

Based on the parameters obtained above, we next perform performance tests on the proposed AFD-based AFTC method. First, the following fault scenarios are considered:

Fault scenarios: The system initially works in a healthy condition; when k∈[160550), the first kind of fault occurs in the system. For k≥550, the previous fault disappears and the second type of fault appears.

**Figure 4:**
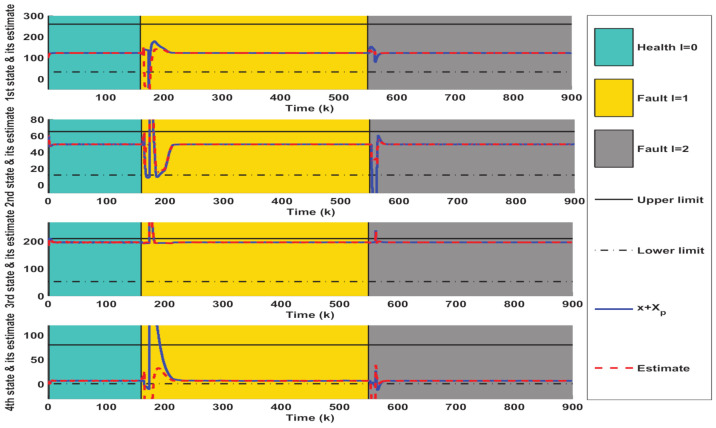
Simulation results of state evolution and estimation under the control of Algorithm 1.

**Figure 5:**
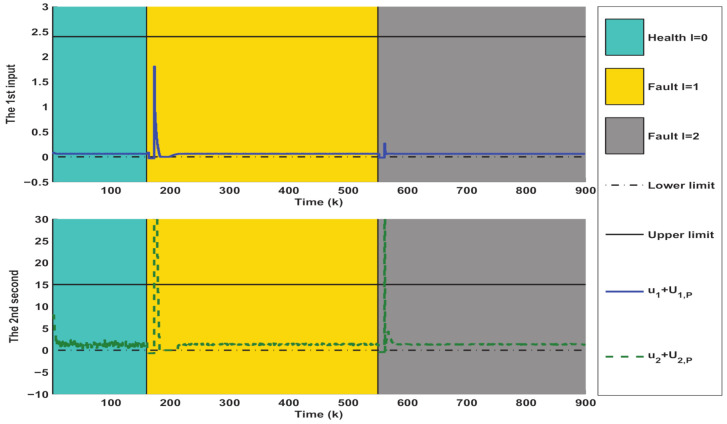
Simulation results of interpolation-based AFTC input obtained from Algorithm 1.

**Figure 6:**
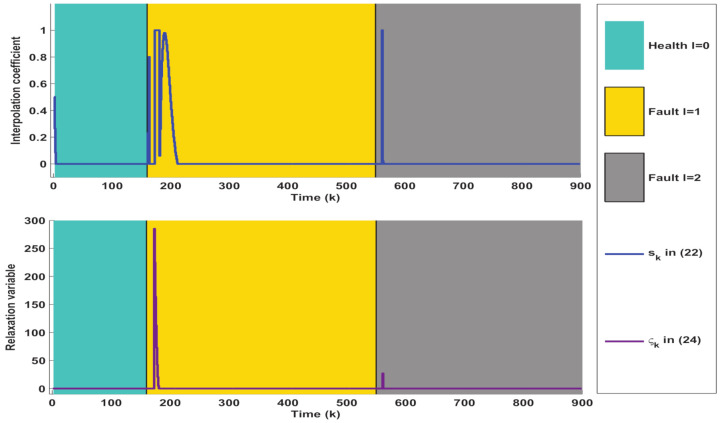
Simulation results of interpolation coefficient and relaxation variable obtained from Algorithm 1.

Then, the online AFTC strategy described in Algorithm 1 is implemented to deal with the above fault situations. The simulation results are collected and depicted in Figure 4, Figure 5 and Figure 6, where the occurrence and duration of different faults have been marked using different color areas, i.e., green area for healthy condition (l=0), yellow area for type I faults (l=1) and gray area for type II faults (l=2). As depicted in Figure 4, it takes some time after a fault occurs to achieve the state regulation to track the equilibrium point $X_{P}$. The reason is that the fault detection, isolation, and control reconfiguration need to be completed during this time. Taking the fault-tolerant process for the first type of fault as an example, Figure 4 firstly depicts that the estimated values of the states can quickly deviate from their actual values in the moments after the fault occurs. Their estimation errors caused by the presence of the fault further generate large residual values, thus facilitating the timely triggering of FI. In fact, the interpolation coefficient in Figure 6 appears to increase rapidly at k>160, which also indicates the occurrence of abnormal system conditions. The inputs of the corresponding constant value auxiliary test signals are further shown in Figure 5. It should be noted that both variables in Figure 6 are zero at this time. After a few steps, it can be seen in Figure 4 that the first three states have been accurately estimated, which indicates that the FI is completed. However, the estimation of the fourth state still deviates from the actual value. The reason is that the auxiliary signal injected during FI drives it to a large deviation (as shown in Figure 2). Hence, additional time is required to achieve its unbiased tracking.

After FI, the corresponding control reconfiguration is further activated. As shown in Figure 6, the soft constraint FTC (24) is triggered first, which also leads to a sharp increase of the control input in Figure 5. When the states are adjusted into $P_{M}^{1}$ by the soft constraint FTC, the interpolation FTC (22) is activated timely. At the same time, as illustrated in Figure 5, the control inputs subsequently become smaller. The decreasing interpolation coefficient in Figure 6 also indicates that the system states are gradually tuned into $\Omega_{MRPI}^{1}$. After that, the states are gradually regulated to track the equilibrium point.

**Remark** **8.** *The above process constitutes a complete cycle of AFD and AFTC. Clearly, the decreasing interpolation coefficients and relaxation variables in Figure 6 fully illustrate the convergence of the proposed Algorithm 1. Correspondingly, the state and control variables in Figure 4 and Figure 5 are also adjusted to the equilibrium point $\left(X_{P},\;U_{P}\right)$, which further illustrates that the control system under the influence of the fault is stabilized and the tracking target is achieved.*

### 4.4. Multi-Performance Comparison and Discussion of Active Fault-Tolerant Control Methods

Some qualitative comparisons with the recently reported AFTC methods are given in Table 1.

The involved comparisons in Table 1 are explained from the following aspects. Firstly, as shown in the second row of Table 1, both component faults and actuator faults are considered in this paper, while only actuator faults are considered in [13,16,20]. In general, the component faults can significantly affect the system dynamics. In this paper, an AFD method is embedded to identify the system operating mode in real time in order to achieve fault tolerance for component faults. Secondly, unlike the multiple-observers-based real-time diagnosis approach in [16], here only one observer needs to be employed at each moment to achieve fault mode identification. Theoretically, this facilitates the diagnosis efficiency and it is also another implicit advantage of using AFD.

In terms of the design and implementation of fault-tolerant methods (i.e., rows 5–7 in Table 1), the MPC optimization problems in [13] are constructed by relying on ellipsoidal constraint sets and LMI, which belongs to SDP and whose solution tends to be more time-consuming. In addition, approximating the feasible domain with ellipsoidal sets is generally more conservative than polyhedral sets. In [20], the dual-mode prediction mechanism is adopted to construct a predictive FTC, whose optimization problem belongs to QP and can be solved relatively efficiently. However, this FTC method is only used to handle actuator additive offset faults and is not suitable for addressing fault tolerance problems of multiplicative faults and component faults. Relatively, the receding horizon set theoretic FTC method in [16] is appealing. This method provides a way to perform the state figure using switching *M*-step controllable ellipsoidal sets under different fault conditions. However, it may be computationally demanding and takes up a large storage space because of the need to solve real-time QP when the states do not belong to the corresponding maximum allowable invariant set. In this paper, the interpolation methods are employed to combine *M*-step controllable polyhedral sets and inner feedback control laws to achieve the state figure, and the corresponding fault-tolerant optimization is formed as LP. Compared to the sets that need to be stored by the FTC method in [16], Algorithm 1 only needs to store the maximum *M*-step controllable polyhedral set for each operating condition, which helps to reduce the storage burden.

The penultimate row of Table 1 illustrates that the soft-constrained FTC method is further integrated into Algorithm 1 and used to deal with some unanticipated situations, such as uncertain fault amplitudes, system parameter drifts, disturbance overruns, etc. The last row of Table 1 implies that the design of the FTC method in [13] is more intuitive and better scalable than the FTC methods in Algorithm 1,^16^,^20^]. It should be noted that the above comparisons are discussed mainly for the characteristics of the involved fault-tolerant methods and not for the contents of the overall studies in [13,16,20]. Clearly, they have different system models and control objectives, and therefore different innovations.

**Remark** **9.** *According to Remark 7, the FTC law based on dual-mode predictive control constructed in [20] can be considered as a special form of the interpolation AFTC developed in this paper. Hence, the interpolation-based AFTC theoretically has a higher degree of design freedom as well as a more efficient optimization capability. To verify this, a further numerical comparison was made. Let the system operate sequentially in two scenarios: scenario I (health l=0) for 1≤k<160 and scenario II (fault l=2) for 160≤k≤500. To be fair, the same active fault diagnosis and integration design were used. Table 2 gives the comparisons of these two methods in terms of interval cost function (3) and running time. It can be seen that the interpolation-based AFTC method runs faster and provides better tracking accuracy for scenario I. In scenario II, the developed interpolation-based AFTC remains feasible and continues to optimize the cost function, however the FTC method of [20] will no longer be feasible after k=170. Based on the above numerical comparisons, the effectiveness of the method constructed in this paper can be further verified.*

## 5. Conclusions

In this paper, a novel activate fault tolerant control scheme is proposed to address the component/actuator faults for the uncertain systems with state/input constraints. Its significant merits are that (1) it relies on only one diagnostic observer for online fault detection and isolation, which helps to reduce the internal memory consumption of the hardware controller; (2) the auxiliary inputs and separation hyperplanes for fault isolation are designed offline in advance, which helps to reduce the online computational burden and increase the freedom of fault isolation decisions; (3) the overall fault tolerant control is reconfigured by optimizing the interpolation coefficient to dynamically regulate the convex combination of inner and outer fault tolerant control laws, which can further reduce the online optimization effort; (4) the inner fault tolerant control and the diagnostic observer are designed offline in advance, and by such design the robust interaction influence on the feasibility of the reconfigured fault tolerant control algorithm can be reduced; (5) the soft constraint method is embedded to achieve a relaxed fault tolerance, which can handle some cases that lead to infeasible constrained optimization in an emergency. The simulation with detailed discussions is given to demonstrate the above benefits of the proposed method.

Some issues need to be further addressed in the future work. For instance, the application of semi-active fault diagnosis to enhance the design flexibility of auxiliary signals; the combination of soft constraint theory and period invariant sets to construct an outer fault tolerant control with flexible and adjustable feasible domains; the construction of parametrization method of interpolated coefficient to avoid solving linear programming problems, etc.

## Figures and Tables

**Table 1:** Performance comparison of different FTC methods.

	Method	Algorithm 1	[20]	[13]	[16]
Performance					
Types of faults that can be handled		Component/actuator faults	Actuator fault	Actuator fault	Actuator fault
Can active fault diagnosis be realized		Yes	-	-	-
Number of observers used in real time		1	-	-	3
Design principle of fault tolerant control		$P_{M}$-based IC	Dual-mode MPC	LMI-based MPC	$P_{M}$-based MPC
Expression of fault tolerant feasible domain		Polyhedral set	Polyhedral set	Ellipsoidal set	Ellipsoidal set
Optimization problems to be solved		LP	QP	SDP	QP
Can active constraint relaxation be achieved		Yes	-	-	-
Extensibility of FTC method		General	General	High	General

Note: interpolating control (IC), model predictive control (MPC), linear matrix inequality (LMI), linear programming (LP), quadratic programming (QP), semi-positive definite programming (SDP), *M*-step robust control invariant set ($P_{M}$).

**Table 2:** Comparisons of interval cost function (3) and running time.

Scenario	Performance	[20]	Interpolation-Based AFTC
Scenario I	Running time	33.2890 (s)	24.6050 (s)
	Evaluation of (3)	128.8576	81.7340
Scenario II	Running time	Infeasible for k≥170	126.7660 (s)
	Evaluation of (3)	-	359.1910

## Data Availability

Not applicable.

## Appendix A

The approximated calculation method of $R_{FI}^{l,i}$ is given below.

By [31], $R_{FI}^{l,i}$ can be explicitly represented as $R_{FI}^{l,i}=\left\{C_{\chi }{\left(I-A_{\chi }^{l,i}\right)}^{-1}B_{\chi }^{l,i}u_{FI}^{i}\right\}⊕C_{\chi }O_{\chi ,\infty }^{l,i}⊕D_{\chi }E$, where $O_{\chi ,\infty }^{l,i}=\left\{\chi :\sum_{j=0}^{\infty }{\left(A_{\chi }^{l,i}\right)}^{j}E_{\chi }^{i}\sigma_{k},\;\sigma_{k}\in E\right\}$. Generally, $O_{\chi ,\infty }^{l,i}$ is difficult to determine, especially for high-dimensional systems. In [27], an external approximation method is proposed to enable $O_{\chi ,\infty }^{l,i}\subseteq \left(1+\mu_{T}\right)O_{\chi ,T}^{l,i}$, where $O_{\chi ,T}^{l,i}=\left\{\chi :\sum_{j=0}^{T}{\left(A_{\chi }^{l,i}\right)}^{j}E_{\chi }^{i}\sigma_{k},\;\sigma_{k}\in E\right\}$ can be calculated in a finite time. Then, for given $u_{FI}^{i}$, the internal point of residual limit set $R_{FI}^{l,i}$ can be parameterized as $C_{\chi }{\left(I-A_{\chi }^{l,i}\right)}^{-1}B_{\chi }^{l,i}u_{FI}^{i}+\left(1+\mu_{T}\right)\sum_{j=0}^{T}C_{\chi }{\left(A_{\chi }^{l,i}\right)}^{j}E_{\chi }^{i}\sigma_{1,k}+D_{\chi }\sigma_{2,k}$, $\forall \sigma_{1,k},\sigma_{2,k}\in E$.

## Appendix B

The proof of **Theorem 1** is given below.

**Proof.** Let $V_{1,k}^{l}=e_{x^{l},k}^{T}P_{1}^{l}e_{x^{l},k}$ and $V_{2,k}^{l}={\left(x_{k}^{l}\right)}^{T}{\left(P_{2}^{l}\right)}^{-1}x_{k}^{l}$ be the Lyapunov functions of (5), (13) and (14), respectively. Equivalently, $V_{1,k}^{l}+V_{2,k}^{l}$ is a Lyapunov function of (15). Define $K^{l}{\left(Y_{2}^{l}\right)}^{T}={\bar{K}}^{l}$ and $Y_{1}^{l}L^{l}={\bar{L}}^{l}$. Then, by using $-Y_{1}^{l}{\left(P_{1}^{l}\right)}^{-1}{\left(Y_{1}^{l}\right)}^{T}\leq P_{1}^{l}-Y_{1}^{l}-{\left(Y_{1}^{l}\right)}^{T}$ and congruence transformation $diag\{I,I,{\left(Y_{1}^{l}\right)}^{-1}\}$ to (16a), the inequality $-diag\left\{P_{1}^{l}-I,\alpha^{2}I\right\}+{\left[\begin{array}{cc} {\tilde{A}}_{11}^{l} & {\tilde{B}}_{1}^{l} \end{array}\right]}^{T}P_{1}^{l}\left(*\right)<0$ can be deduced. It further implies that the relation $V_{1,k+1}^{l}-V_{1,k}^{l}+e_{x^{l},k}^{T}e_{x^{l},k}-\alpha^{2}\varrho_{k}^{T}\varrho_{k}<0$ holds. Similarly, by using the inequality $-Y_{2}^{l}{\left(P_{2}^{l}\right)}^{-1}{\left(Y_{2}^{l}\right)}^{T}\leq P_{2}^{l}-Y_{2}^{l}-{\left(Y_{2}^{l}\right)}^{T}$ and congruence transformation factor $diag\{{\left(Y_{2}^{l}\right)}^{-1},{\left(Y_{2}^{l}\right)}^{-1},I,I,I\}$ to (16b), we get the inequality $-diag\left\{{\left(P_{2}^{l}\right)}^{-1},{\left(P_{2}^{l}\right)}^{-1},\beta^{2}I\right\}+{\left[\begin{array}{ccc} {\tilde{A}}_{21}^{l}\; & \;{\tilde{A}}_{22}^{l}\; & \;{\tilde{B}}_{2}^{l} \end{array}\right]}^{T}\;{\left(P_{2}^{l}\right)}^{-1}\;\left(*\right)\;+\;{\left[\begin{array}{ccc} {\bar{D}}^{l}\; & \;{\bar{C}}^{l}\; & \;0 \end{array}\right]}^{T}\left(*\right)<0$. Based on the Lyapunov function $V_{2,k}^{l}$ and the system model (13) and (14), we can further derive $V_{2,k+1}^{l}-V_{2,k}^{l}+{\left(z_{k}^{l}\right)}^{T}z_{k}^{l}-e_{x^{l},k}^{T}{\left(P_{2}^{l}\right)}^{-1}e_{x^{l},k}-\beta^{2}\varrho_{k}^{T}\varrho_{k}<0$. Under zero initial conditions, the summation of $V_{2,k+1}^{l}-V_{2,k}^{l}+{\left(z_{k}^{l}\right)}^{T}z_{k}^{l}-e_{x^{l},k}^{T}{\left(P_{2}^{l}\right)}^{-1}$$e_{x^{l},k}-\beta^{2}\varrho_{k}^{T}\varrho_{k}<0$ over k=0 to k=∞ can be bounded in the form of $\|z_{k}^{l}{\|}_{2}^{2}\leq \epsilon^{2}\|e_{x^{l},k}{\|}_{2}^{2}+\beta^{2}{\left\|\varrho_{k}\right\|}_{2}^{2}$, where $e_{x^{l},k}^{T}{\left(P_{2}^{l}\right)}^{-1}e_{x^{l},k}$ is relaxed by $e_{x^{l},k}^{T}{\left(P_{2}^{l}\right)}^{-1}e_{x^{l},k}\leq eig_{max}\left({\left(P_{2}^{l}\right)}^{-1}\right)$$e_{x^{l},k}^{T}e_{x^{l},k}=\epsilon^{2}e_{x^{l},k}^{T}e_{x^{l},k}$. Similarly, $\sum_{k=0}^{\infty }\left\{V_{1,k+1}^{l}-V_{1,k}^{l}+e_{x^{l},k}^{T}e_{x^{l},k}-\alpha^{2}\varrho_{k}^{T}\varrho_{k}\right\}<0$ leads to $\|e_{x^{l},k}{\|}_{2}^{2}\leq \alpha^{2}{\left\|\varrho_{k}\right\|}_{2}^{2}$ under zero initial conditions. Then, we further have $\|z_{k}^{l}{\|}_{2}^{2}\leq \left(\epsilon^{2}\alpha^{2}+\beta^{2}\right){\left\|\varrho_{k}\right\|}_{2}^{2}$. Clearly, the integrated optimization of α and β contributes to improving the synthesized $H_{\infty }$ performances of observer (5) and unconstrained robust control policy $u_{C,k}^{l}=K^{l}{\hat{x}}_{k}^{l}$. The proof is completed. □
